# Current Orthodontic Education Status on Treating Patients With ASD and/or ADHD in North America

**DOI:** 10.1002/jdd.70091

**Published:** 2025-10-24

**Authors:** Nikki Wei, Chun‐Hsi Chung, Ilana Zinn, Mark S. Wolff, Chenshuang Li

**Affiliations:** ^1^ Department of Orthodontics School of Dental Medicine University of Pennsylvania Philadelphia Pennsylvania USA; ^2^ Johns Hopkins School of Medicine Baltimore Maryland USA; ^3^ Division of Restorative Dentistry School of Dental Medicine University of Pennsylvania Philadelphia Pennsylvania USA

**Keywords:** attention‐deficit hyperactivity disorder, autism spectrum disorder, orthodontics, postgraduate education, survey

## Abstract

**Objectives:**

Autism spectrum disorder (ASD) and attention‐deficit hyperactivity disorder (ADHD) are associated with specific dental and behavioral attributes, particularly in children, that necessitate special attention from healthcare providers. This study examines the training provided in postgraduate orthodontic programs in North America regarding the treatment of patients with ASD and/or ADHD.

**Methods:**

A multiple‐choice online survey was sent via Qualtrics requesting the anonymous participation of program directors/chairs of all 75 postgraduate orthodontic programs in North America.

**Results:**

The survey response rate was 38.7% with 29 complete responses. Of the respondents, only 6.9% (two) had specific protocols to treat these patients in their postgraduate clinics. Most respondents did not teach about malocclusion differences (72.4%), other dental differences (55.2%), patient behavior management strategies (37.9%), or guardian communication strategies (65.5%) for patients with ASD and/or ADHD. Of the 20 programs that treat patients with ASD and/or ADHD in their postgraduate clinics, 60.0% of the respondents reported that they do not have specific requirements for faculty member(s) who provide clinical instructions on managing these patients. For the 13 programs that offer lectures on treating patients with ASD and/or ADHD, predominantly faculty member(s) with a craniofacial fellowship or faculty member(s) with more clinical experience in this area give the lectures.

**Conclusion:**

Few North American postgraduate orthodontic programs provide systematic education about treating patients with ASD and/or ADHD. There needs to be more consistency in the curriculum content. A standardized and systematic education curriculum is necessary to better prepare postgraduate trainees for treating children with ASD and/or ADHD.

## 1 Introduction

The prevalence of autism spectrum disorder (ASD) and attention deficit‐hyperactivity disorder (ADHD) has increased significantly in recent years [1, 2, 3]. This rise is likely due to a combination of factors, including maternal obesity, preterm birth, increased public awareness, better screening, and diagnostic tools, and potentially, changes in diagnostic criteria [4, 5]. These conditions may present with characteristic traits that influence how patients experience healthcare and how health professionals approach the delivery of care. Although ASD and ADHD are distinct diagnoses, there are some intersections in dental and behavioral attributes that translate into overlaps in strategies for dental care. For example, children with ADHD have an elevated risk for dental trauma and caries [6, 7, 8]. Both ASD and ADHD are associated with a greater risk for periodontal disease [7, 9]. Both patient populations have been reported to experience difficulties maintaining oral hygiene and benefit from the additional support of parents/caregivers and the dental team [10, 11]. Thus, proper dental condition monitoring and oral hygiene control are critical during dental care for this population.

In addition, ASD and ADHD are linked to a higher prevalence and severity of malocclusion [12, 13, 14, 15], which calls for potential orthodontic care. Some observed malocclusion trends include narrow and deep palate, anterior open bite, increased overjet, posterior crossbite, lip incompetence, severe crowding, and increased risk for Class I and II malocclusion [7, 8, 13, 15, 16, 17]. This increased risk for malocclusion may be related to parafunctional habits like bruxism, tongue thrusting, object biting, and mouth breathing, which have been previously reported in individuals with ASD and/or ADHD [7, 8, 14, 17, 18, 19]. This propensity towards oral diseases combined with difficulty finding a dental home can cause oral conditions and habits to increase in severity due to a lack of early treatment [13, 19]. As a result, many of these patients end up requiring emergency treatments which may have been prevented by routine dental visits at an earlier age [19]. Early dental visits and orthodontic intervention are recommended by some clinicians for this patient population to avoid worsening malocclusion and to improve oral health and overall quality of life [12, 13, 18, 20].

In addition to the risk for oral conditions, there are also behavioral characteristics associated with ASD and/or ADHD that require specific clinical management techniques. In the dental practice environment, even routine procedures can be overwhelming and invasive to patients with ASD [19, 21]. Multiple studies have reported that individuals with ASD experience hypersensitivity and may be exceptionally perceptive to noises, sounds, smells, and sensations associated with the dental environment, such as an exaggerated gag reflex and fear of sharp instruments commonly found in the operatory [8, 12, 22, 23, 24]. For dental patients with ADHD, providers should be understanding of lateness, prioritize early morning appointments, and warn parents/caregivers of increased risk for trauma [9, 11]. Patients with ASD and/or ADHD are also less comfortable remaining still for long periods, exhibit shorter attention spans, and are more likely to experience dental anxiety [23, 25]. Recommendations for the effective delivery of care to patients with ASD and/or ADHD include discussing the patient with patients/parents/caregivers for best approaches, desensitization visits, maintaining continuity and consistency in staff and operatories, developmentally appropriate communication, positive reinforcement, scheduled breaks during treatment, visual pedagogy, and use of the “tell‐show‐do” technique [8, 26, 27, 28]. While many of the recommended behavior management strategies are not exclusive to the treatment of patients with ASD and/or ADHD, these specific approaches have been found to be especially helpful for this patient population.

Effective communication with patients’ parents/guardians is another critical component of orthodontic care, as household support is crucial in this patient population. For instance, it can be essential to enlist the patient's family's help to promote oral hygiene during orthodontic treatment [19, 25]. It has been noted that the parents of children with ASD and/or ADHD emphasize improved function over esthetics as a priority for orthodontic treatment [12]. Understanding the goals and concerns of the patients and guardians can be helpful for treatment planning and motivating the patients during treatment. Incorporating caregivers in dental treatment and emphasizing collaboration between the patient, guardians, and dental team can be instrumental in successfully treating patients with ASD [19]. Studying behavioral guidance recommendations and learning strategies for communication with the patients and their guardians can aid the provider in navigating appointments and treating this patient population knowledgeably and compassionately.

There is an ongoing debate about whether patients with ASD and/or ADHD should receive different treatment compared to other patients. However, previous research has highlighted differences in malocclusion tendencies, various dental trends, and behavioral characteristics that must be considered. Therefore, it is essential to adequately prepare orthodontic residents during their training to ensure that they can provide appropriate care for the increasing population of patients with ASD and/or ADHD. There is research suggesting that clear aligner therapy is beneficial for patients with ASD [29], but currently, no studies have been published directly comparing clear aligners versus fixed appliances in patients with ASD and/or ADHD. Integrating a variety of techniques and tailoring treatment accordingly as the provider–patient relationship develops is essential for the inclusive and effective treatment of patients with ASD and/or ADHD [19].

The Commission on Dental Accreditation (CODA) has developed standards for an advanced fellowship program—a subspecialty of Orthodontics and Dentofacial Orthopedics—to provide proper training to orthodontists who desire to provide care to patients with craniofacial syndromes and/or need special care. Currently, there are seven Craniofacial and Special Care Orthodontics Fellowship programs that are CODA‐accredited and several programs that are not CODA‐accredited in the United States and Canada. Unfortunately, while orthodontic residents acknowledge the importance of treating patients with craniofacial anomalies, cleft lip/palate, and special needs, only 29.3% of residents report that they would complete a 1‐year fellowship [30]. The limited number of orthodontists trained by fellowship programs cannot meet the increasing demand for orthodontic care of the increasing population of children with ASD and/or ADHD. In addition, it is worth noting that almost all craniofacial orthodontic fellowship programs are based in hospital settings, which limits the direct adoption and implementation of curriculum components by orthodontic postgraduate programs. Even if graduate trainees are able to experience care in hospital settings, it might be difficult to directly apply what they learned to a regular orthodontic office setting that does not have easy access to the comprehensive supports of the medical teams. Thus, proper training in regular, post‐graduate orthodontic residency programs becomes crucial for preparing orthodontic postgraduate trainees to provide proper care to patients with special needs. The purpose of this study is to determine the current education status of post‐graduate orthodontic residency programs in the United States and Canada on the topic of treating patients with ASD and/or ADHD.

## 2 Methods

A multiple‐choice survey was created using Qualtrics online software (Provo, UT, USA). The survey included questions intended to provide a broad overview of the didactic and clinical trainings currently offered by North American postgraduate orthodontic programs on the topic of treating patients with ASD and/or ADHD. The questionnaire was designed to gather information on ASD and/or ADHD‐related curriculum content, the faculty member(s) providing instruction on these topics, and how this content was presented to postgraduate trainees. To cover all the information mentioned above, the questions were set to include each step of clinical care (ranging from diagnosis, clinical treatment, patient behavior management, and communication with the guardians), type of teaching (lecture, seminar, case simulation, and clinical experience), as well as resources for teaching (teaching clinic settings, and faculty member[s]). The topics for these questions were selected by reviewing previous studies and identifying recurring subjects related to patient care for those with ASD and/or ADHD [8, 11, 12, 21, 22, 23, 24, 26, 27]. The survey consisted of 18 multiple‐choice questions, all written in English. Follow‐up questions were programmed to appear based on the responses given. The questionnaire was structured in five blocks, namely: malocclusion differences, other dental differences, patient behavior management, guardian communication, and teaching resources. There were three questions in each of the first four blocks, and six questions in the last block. For specific questions, respondents could select “Other” and provide a written answer if none of the provided choices were applicable. In addition, some questions allowed respondents to select multiple answers if relevant. The survey was validated through pilot tests conducted by three full‐time orthodontic faculty members with different levels of exposure to special needs. For instance, there was one faculty member with a craniofacial orthodontic fellowship training background, one faculty member who does not have a craniofacial orthodontic fellowship training background but was extensively exposed to orthodontic care for patients with special needs during their residency training, and one faculty member who does not have a craniofacial orthodontic fellowship training background but works closely with orthodontists in a children's hospital with proper referral protocol. The three faculty members were asked if any question in the questionnaire or the sequence of the questions appearing in the questionnaire led to potential confusion. In addition, there were two faculty members teaching in the same institute. Their responses to the questionnaire were compared, and a 100% agreement was achieved.

As this survey aimed to collect program and facility information, approval from the University of Pennsylvania Institutional Review Board (IRB) was exempted after consultation with the IRB. The IRB exemption status was stated in the survey invitation to notify all the invited participants that “The current project is exempted from the University of Pennsylvania Institutional Review Board (IRB) approval. This exemption may or may not be sufficient at your local institution. Should you have any questions or concerns, please feel free to contact us.*”*

The email contact information of the program director and department chair for each orthodontic postgraduate program was obtained on the website of the American Association of Orthodontists (https://www2.aaoinfo.org/programs‐for‐residents‐and‐educators/accredited‐orthodontic‐programs/), and further validated by checking the websites of each program. On April 10th, 2023, the survey was sent to the program directors of all 75 North American postgraduate orthodontic programs. Following this initial invitation, two reminder emails were sent in 2‐week intervals to the program directors who still needed to complete the survey. On May 30th, 2023, the survey invitation was emailed to the department chairs of the programs whose program directors had not submitted the survey. The same follow‐up email schedule was used with the program chairs, with two reminder emails sent every 2 weeks to the program chairs who still needed to complete the survey yet. As the responses were recorded anonymously, all follow‐up emails were programmed to be sent automatically through Qualtrics software. The survey was closed on June 26th, 2023. The responses were reviewed on Qualtrics to remove any incomplete responses. Data was then transferred to the GraphPad Prism (version 8.2.1, San Diego, CA, USA) for further analysis, visualization, and figure generation.

## 3 Results

The complete response rate for this survey was 38.7%, with 29 complete responses received out of the invitations to 75 programs. The regional distribution of the 29 responses are: two belonging to the Great Lakes Association of Orthodontist, five belonging to the Middle Atlantic Society of Orthodontists, four belonging to the Midwestern Society of Orthodontists, five belonging to the Northeastern Society to Orthodontists, four belonging to the Pacific Coast Society of Orthodontists, zero belonging to the Rocky Mountain Society of Orthodontists, six belongs to the Southern Association of Orthodontists, and three belonging to the Southwestern Society of Orthodontists. Due to the large variations in the numbers of responding programs among each regional constituent group, statistical analysis comparing different regions was not performed.

### 3.1 Clinical Protocol

**FIGURE 1:**
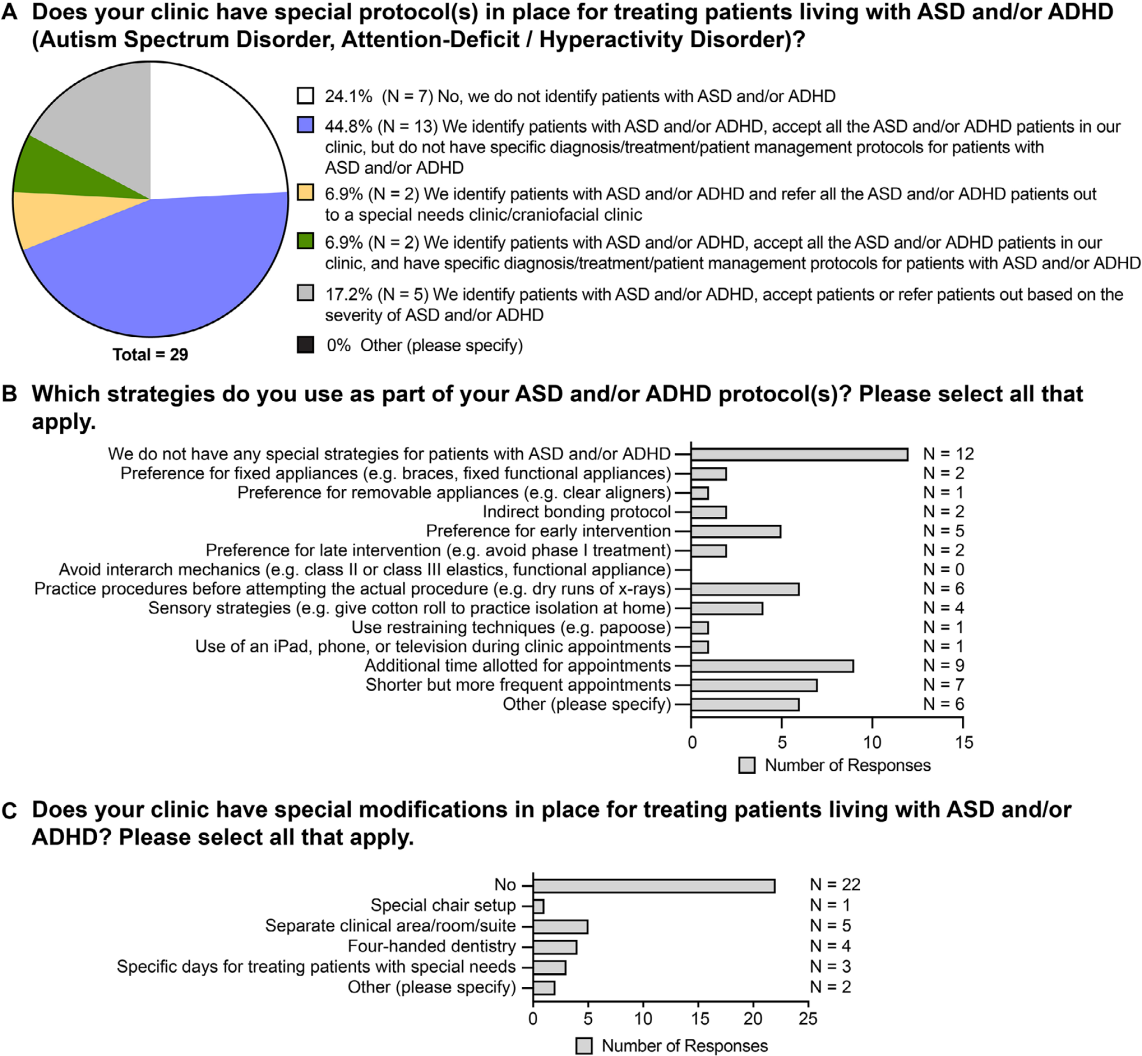
Clinical protocols for treating patients with ASD and/or ADHD. (A) Responses to the question “Does your clinic have special protocol(s) in place for treating patients living with ASD and/or ADHD?” (B) Responses to the question “Which strategies do you use as part of your ASD and/or ADHD protocol(s)? Please select all that apply.” (C) Responses to the question “Does your clinic have special modifications in place for treating patients living with ASD and/or ADHD? Please select all that apply.”

When asked if the programs had specific clinical protocol(s) in place for treating patients with ASD and/or ADHD, 6.9% (two responses) of the respondents reported that they identified patients with ASD and/or ADHD, accepted them into their postgraduate clinic, and had specific protocols in place to treat them (Figure 1A). The other respondents either identified and accepted patients with ASD and/or ADHD but did not have specific protocols to treat them (44.8%, 13 responses), did not identify (24.1%, seven responses), identified and referred to other clinics based on severity of ASD and/or ADHD (17.2%, five responses), or identified and referred all of these patients out to a special needs clinic/craniofacial clinic (6.9%, two responses) (Figure 1A).

For the programs that identify patients with ASD and/or ADHD, they were also asked to specify the strategies utilized in their clinics as part of their ASD and/or ADHD protocol(s) (Figure 1B). Respondents could select multiple options from a list of techniques or choose “other” to manually enter a response. This question was also set to validate the consistency of responses obtained from each respondent further. Twelve programs (41.4%) indicated that they did not employ any particular strategies for treating patients with ASD and/or ADHD, and the other programs selected a range of techniques, with “additional time allotted for appointment” and “shorter but more frequent appointments” as the top two chosen options (Figure 1B). Most respondents who selected “other” indicated that each case is different, and the clinical management and treatment are based on the individual's needs. In addition, one respondent pointed out that providing a cotton roll for desensitization at home constitutes a choking hazard to either the patients or other children/persons in the home environment and would not be provided by their clinic. However, some other programs have suggested this strategy.

For the programs that identify patients with ASD and/or ADHD, we also inquired about any special clinical modifications that programs used to treat patients with ASD and/or ADHD (Figure 1C). Most respondents stated that they do not have special modifications in place for treating patients living with ASD and/or ADHD (75.9%, 22 responses). Five programs have separate clinical area for patients with ASD and/or ADHD. One respondent stated that although their clinic doesn't have special modifications now, they do hope to equip one operatory as a sensory‐adapted dental environments (SADE) room in near future per the American Academy of Pediatric Dentistry (AAPD) reference manual (https://www.aapd.org/globalassets/media/policies_guidelines/bp_behavguide.pdf).

### 3.2 Teaching on Malocclusion Difference

**FIGURE 2:**
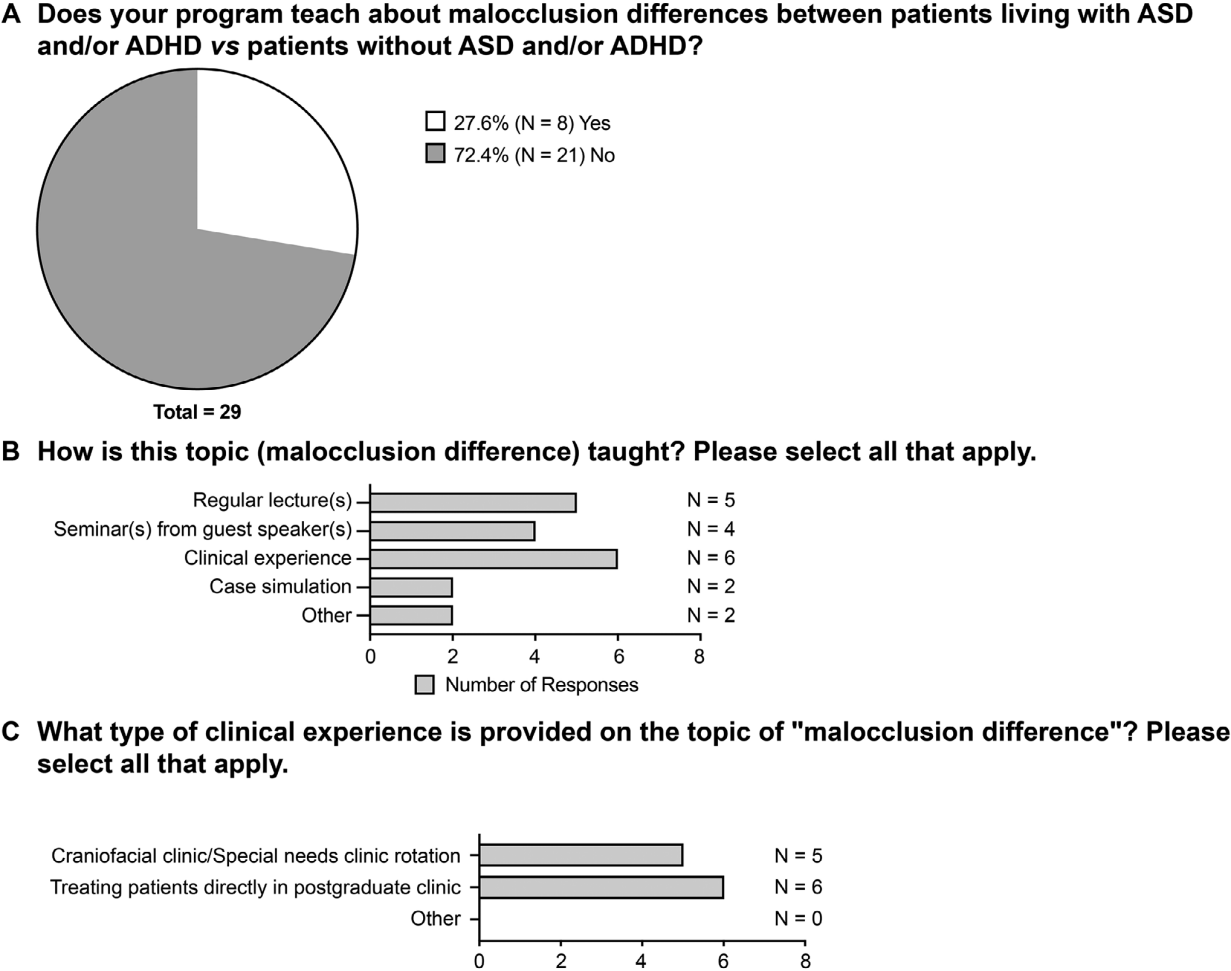
Curriculum content on malocclusion differences between patients living with ASD and/or ADHD versus patients without ASD and/or ADHD. (A) Responses to the question “Does your program teach about malocclusion differences between patients living with ASD and/or ADHD versus patients without ASD and/or ADHD?” (B) Responses to the question “How is this topic (malocclusion difference) taught? Please select all that apply.” (C) Responses to the question “What type of clinical experience is provided on the topic of ‘malocclusion difference’? Please select all that apply.”

The next section of the survey inquired about curriculum content regarding malocclusion differences between patients living with ASD and/or ADHD versus patients without ASD and/or ADHD. 72.4% (21 responses) of the respondents reported that they did not teach about malocclusion differences between the two groups (Figure 2A). For the eight programs (27.6%) that did include this content in their curriculums, the formats of teaching they have utilized include (Figure 2B) regular lecture(s) (five responses), seminar(s) from guest speaker(s) (four responses), clinical experience (six responses), case simulation (two responses), and two programs selected “other” (one stated the topic is only occasionally taught, and another one stated clinical rotations in advanced care clinic and open discussion among residents and faculty member[s]).

For the six respondents who indicated clinical experience as part of their teaching methods, almost every program has clinical experience provided with treating patients directly in postgraduate clinic and craniofacial clinic/special needs clinic rotation (Figure 2C).

### 3.3 Teaching on Other Dental Differences

**FIGURE 3:**
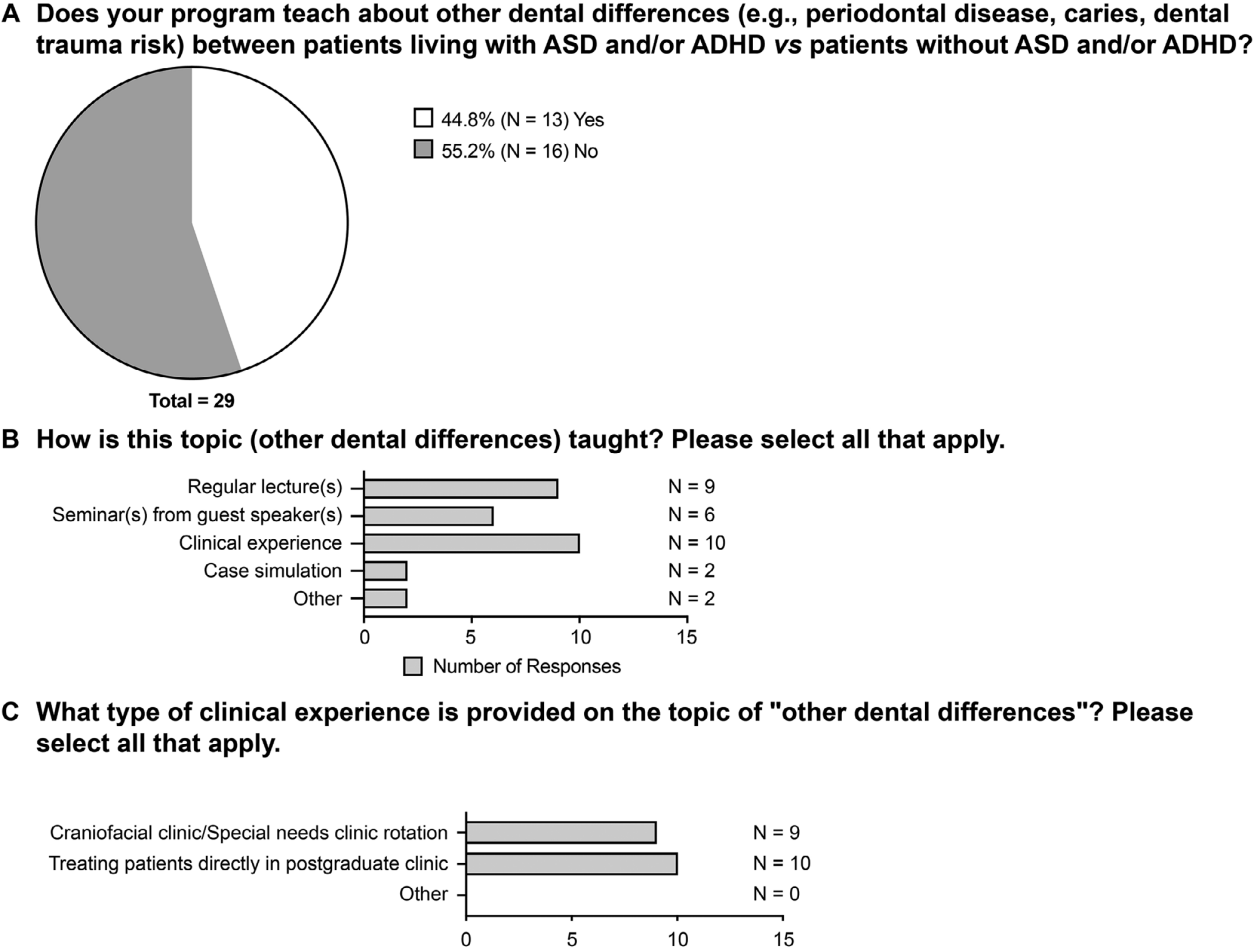
Curriculum content on other dental differences between patients living with ASD and/or ADHD versus patients without ASD and/or ADHD. (A) Responses to the question “Does your program teach about other dental differences (e.g., periodontal disease, caries, dental trauma risk) between patients living with ASD and/or ADHD versus patients without ASD and/or ADHD?” (B) Responses to the question “How is this topic (other dental differences) taught? Please select all that apply.” (C) Responses to the question “What type of clinical experience is provided on the topic of ‘other dental differences’? Please select all that apply.”

Programs were also asked about whether their curriculum included teaching about other dental differences (e.g., periodontal disease, caries, dental trauma risk) between patients with ASD and/or ADHD and those without these conditions. A percentage of 55.2 (16 respondents) indicated that they did not teach about dental differences, while 44.8% (13 respondents) stated they did teach about dental differences (Figure 3A). Among these 13 respondents, there was again a range of ways that this subject was taught. The most common methods selected were through regular lecture(s) (nine respondents) and clinical experience (10 respondents) (Figure 3B). Almost all 10 programs offer clinical experience in treating patients directly, which includes rotations in postgraduate clinics and craniofacial or special needs clinics (Figure 3C).

### 3.4 Teaching on Patient Behavior Management

**FIGURE 4:**
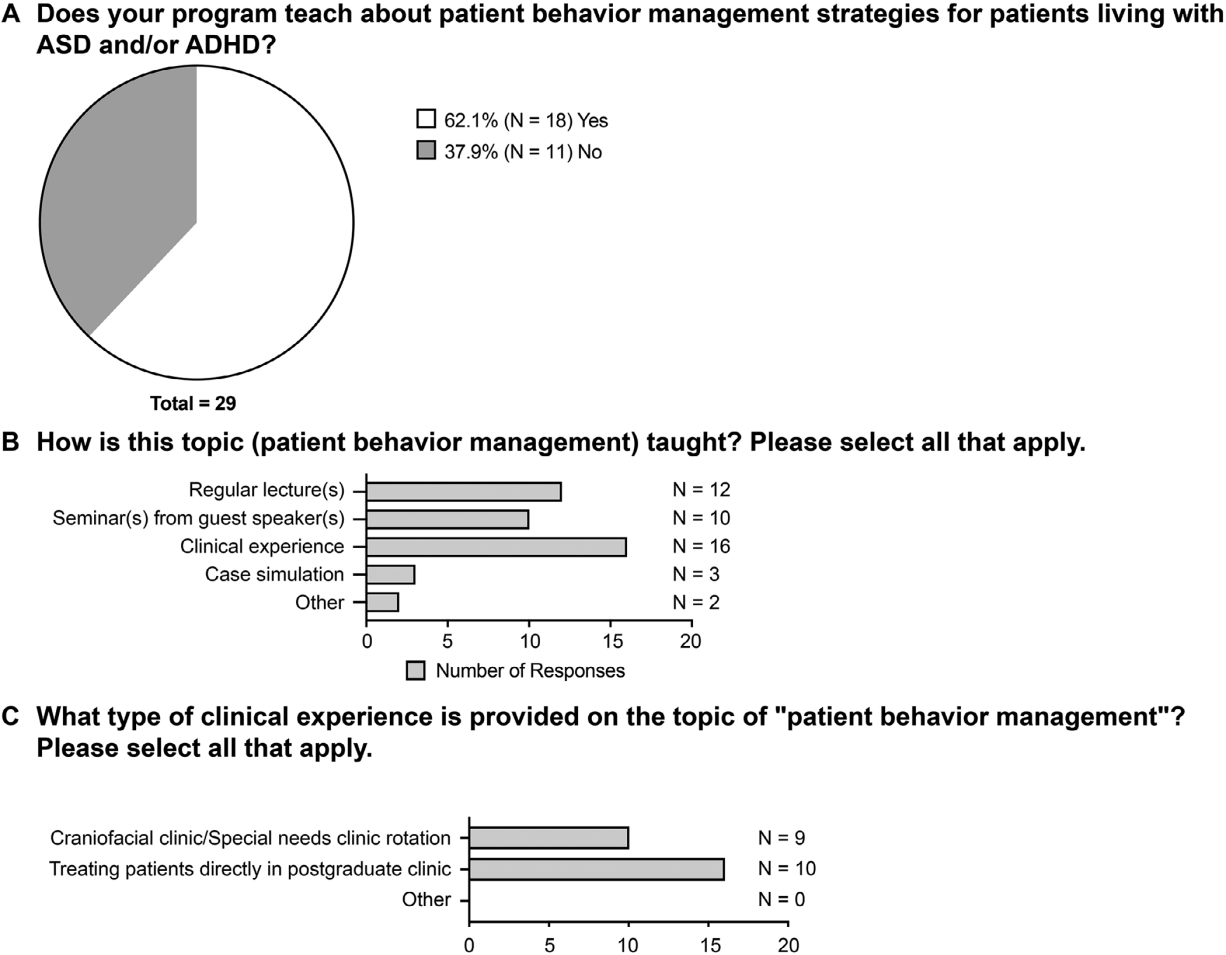
Curriculum content on patient behavior management strategies between patients living with ASD and/or ADHD versus patients without ASD and/or ADHD. (A) Responses to the question “Does your program teach about patient behavior management strategies for patients living with ASD and/or ADHD versus patients without ASD and/or ADHD?” (B) Responses to the question “How is this topic (patient behavior management) taught? Please select all that apply.” (C) Responses to the question “What type of clinical experience is provided on the topic of ‘patient behavior management’? Please select all that apply.”

The third topic we inquired about was teaching on patient behavior management. For this subject, the majority of respondents (62.1%, 18 respondents) shared that they did provide instruction on this topic (Figure 4A). The most common methods of teaching included regular lecture(s) reported by 12 respondents and clinical experience noted by 16 respondents (Figure 4B), with the clinical experiences obtained in both craniofacial clinic/special needs clinic rotations and treating patients directly in the postgraduate clinic (Figure 4C). In addition, seminar(s) featuring guest speaker(s) were also a prevalent method of educating the residents on patient behavior management (Figure 4B).

### 3.5 Instruction on Communication With Parents/Caregivers

**FIGURE 5:**
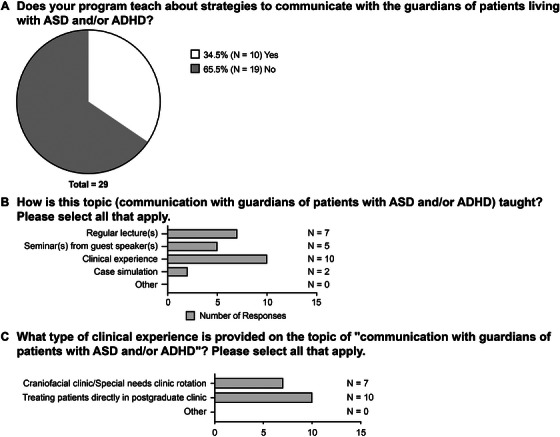
Curriculum content on communication with the guardians of patients living with ASD and/or ADHD versus patients without ASD and/or ADHD. (A) Responses to the question “Does your program teach about strategies to communicate with guardians of patients living with ASD and/or ADHD?” (B) Responses to the question “How is this topic (communication with guardians of patients with ASD and/or ADHD) taught? Please select all that apply.” (C) Responses to the question “What type of clinical experience is provided on the topic of ‘communication with guardians of patients with ASD and/or ADHD?’ Please select all that apply.”

The final curriculum topic we examined was whether programs teach about strategies to communicate with the parents/caregivers of patients with ASD and/or ADHD. A percentage of 65.5 (19 respondents) stated that they did not teach about strategies for guardian communication, while 34.5% (10 respondents) indicated that they did cover this subject (Figure 5A). The majority of respondents (10 respondents) selected clinical experience to describe how this topic was taught in their program, followed by regular lecture(s) (seven respondents) (Figure 5B). The type of clinical experience provided on the topic of communication with guardians of patients with ASD and/or ADHD was most commonly treating patients directly in postgraduate clinic (10 respondents) (Figure 5C).

### 3.6 Faculty Resources of Teaching

**FIGURE 6:**
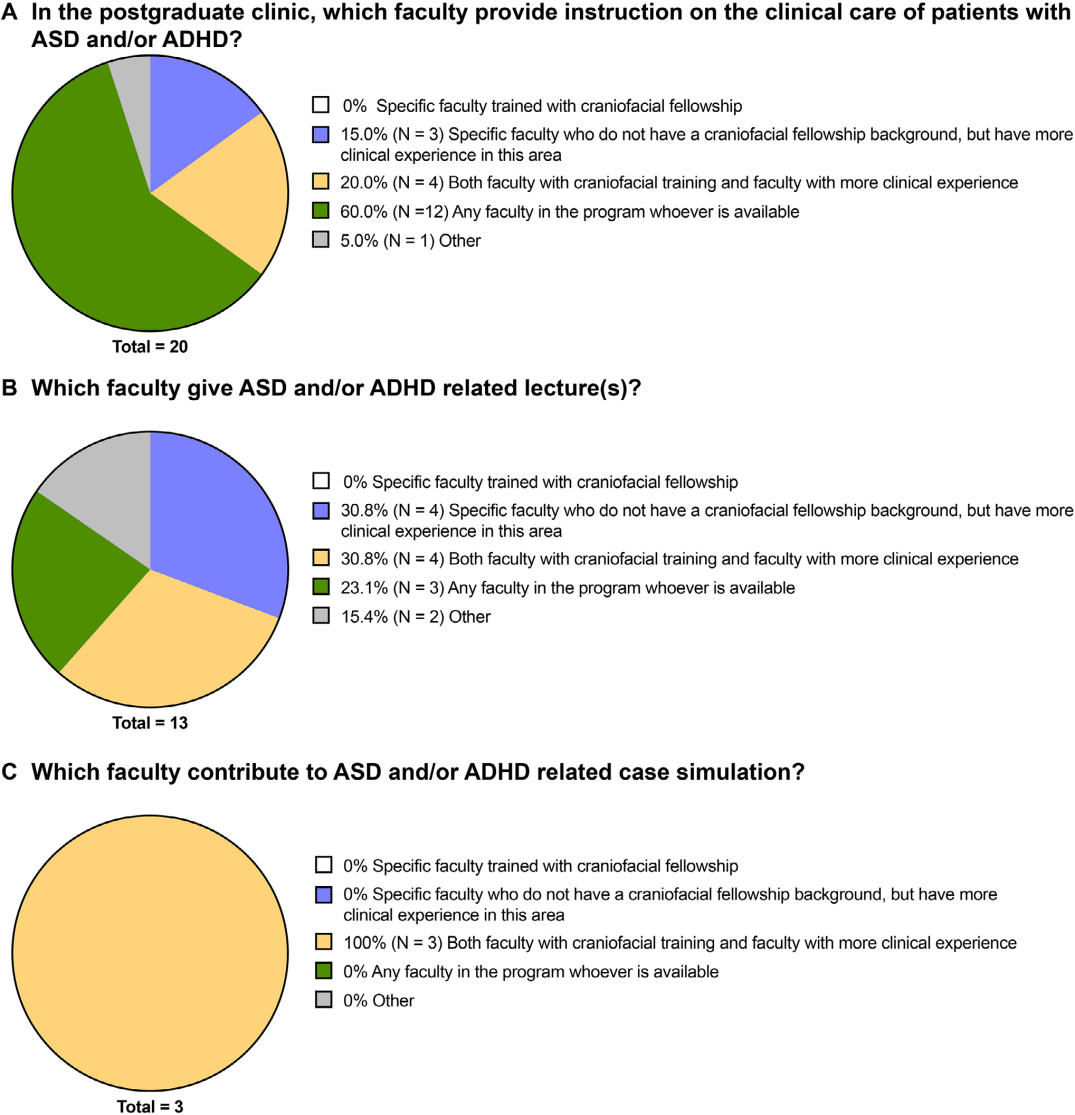
Faculty providing instruction on treating patients with ASD and/or ADHD. (A) Responses to the question “In the postgraduate clinic, which faculty provide instruction on the clinical care of patients with ASD and/or ADHD?” (B) Responses to the question “Which faculty give ASD and/or ADHD related lecture(s)?” (C) Responses to the question “Which faculty contribute to ASD and/or ADHD related case simulation?”

In addition to investigating the content of the postgraduate orthodontic program's curriculum, we also asked about the faculty members(s) who instruct in treating patients with ASD and/or ADHD. For the programs that treat patients with ASD and/or ADHD in their postgraduate clinics (20 respondents), most of these programs indicated that residents go to any faculty member(s) in the program who is available (60.0%, 12 respondents). The other options included faculty member(s) with craniofacial training and faculty with more clinical experience in this area (20.0%, four respondents), specific faculty member(s) who do not have a craniofacial fellowship background but have more clinical experience in this area (15.0%, three respondents), specific faculty member(s) trained with craniofacial fellowship (0%, no respondents), and other (5.0%, one respondent, stated “all faculty member(s) are trained and capable of managing this patient population”) (Figure 6A).

We also asked which faculty member(s) provided ASD and/or ADHD related lecture(s) (Figure 6B). The same answer choices were given. Of the 13 programs that offer lectures on treating patients with ASD and/or ADHD, we saw the most responses for two answer choices: specific faculty member(s) who do not have a craniofacial fellowship background but have more clinical experience in this area (30.8%, four respondents), and both faculty member(s) with craniofacial training and faculty member(s) with more clinical experience (30.8%, four respondents) (Figure 6B). For the two programs that selected “other,” one program stated that “all faculty are trained,” and another program said that “pediatric dentistry faculty.”

The three programs that include ASD and/or ADHD case simulation in their curriculum indicated that faculty member(s) with craniofacial fellowships and faculty member(s) with more clinical experience treating this patient population contributed to the case simulations (Figure 6C).

## 4 Discussion

Demand for orthodontic treatment has increased significantly in the past decades, with the tangentially growing awareness of oral health benefits and increasing desire for aesthetic improvement [31, 32]. An important mission for educators of postgraduate orthodontic residency programs is to train the new generation of orthodontists to provide proper care for this growing patient population. Inclusive treatment for the whole population involves treating patients with special healthcare needs (SHCN), as children with autism often require orthodontic treatment at a higher rate than their neurotypical peers due to a higher prevalence of malocclusion, related dental issue, and higher socio‐psychological need [33, 34]. The global trends in the past few decades reveal a rise in diagnoses of ADHD and ASD that is reflected in the North American population. ADHD is the most common childhood‐onset neurodevelopmental disorder, with 10% of adolescents aged 4–17 years old diagnosed with ADHD in the United States [2]. Studies from the United States and Canada also indicated a significant increase in ASD prevalence amongst children aged 1–17 within the past 20 years [3]. Neurodevelopmental disorders such as ASD and/or ADHD are linked to oral health disturbances, including but not limited to increased periodontal disease, caries risk, and malocclusion tendency, which can all lead to decreased quality of life [10]. Therefore, dental providers need to be able to serve this growing group of individuals and meet their oral healthcare needs. It is worth noting that hypersensitivity associated with individuals living with autism usually makes dental treatment for this population difficult [33]. Serving this population requires appropriate treatment environments, and most importantly, well‐trained specialists who are experienced in providing care to patients with special needs.

Unfortunately, many parents of children with ASD and/or ADHD report difficulty finding dental providers who are willing to treat their children [14, 35]. In a survey administered to general dentists in the US, only 40% of respondents indicated that they would treat children with ASD [36]. Some providers felt that their dental education was insufficient in treating patients with special needs [36]. However, both pediatric and general dentists reported that if they had more training in caring for this patient population, they would be more likely to treat them in their clinics [35, 36]. Since orthodontic care is a critical part of children's and teenagers’ dental care, proper training of orthodontic residents on this topic is essential for providing sufficient care to patients with ASD and/or ADHD. At the moment, there is no CODA requirement for postgraduate orthodontic programs on education for treating patients with special needs. The most relevant statement requires that programs include curriculum on how to “manage patients with intellectual and developmental disabilities.” [37] Interestingly, according to the US Centers for Disease Control and Prevention [38], ASD is a developmental disability while ADHD is categorized under “neurodevelopmental disorders.” Thus, children with ASD and/or ADHD can all be classified as “individuals with special health care needs,” but not necessarily as “patients with disabilities.” The current study includes both populations to investigate the current education provided by orthodontic residencies on treating patients with ASD and/or ADHD.

Most of the programs that responded to our survey reported that while they accept and treat patients with ASD and/or ADHD in their postgraduate clinics, they do not have specific clinical protocols in place to inform treatment (Figure 1A). The absence of standardized guidelines for patients with ASD and/or ADHD seems contrary to findings from previous studies which identify differences in operatory set‐up, behavior guidance, and dental tendencies for patients with ASD and/or ADHD that should be acknowledged when treating this patient population [7, 8, 12, 16, 17]. Even for the programs that indicated that they did have particular protocols in place, it was observed that these strategies could be varied and sometimes contradicting. For example, some programs preferred longer appointments while others favored shorter but more frequent visits, and some programs preferred early intervention while others preferred late intervention (Figure 1B).

Regarding didactic curriculum content, most respondents did not teach about malocclusion differences, other dental differences, or patient/caregiver communication. The only topic we inquired about that most of the programs covered in didactic coursework was patient behavior management. When asked about the faculty member(s) providing instruction on treating patients with ASD and/or ADHD in the clinic setting, the faculty member(s) overseeing care were most likely to be any faculty available. In contrast, for didactic instruction, the faculty member(s) were more likely to be those with a craniofacial fellowship or faculty member(s) who have more experience treating patients with ASD and/or ADHD. The lack of consistency in faculty member(s) assignment could also contribute to variations in lesson content.

In the CODA standard for advanced dental education programs, the requirement for pediatric dentistry is stated as: “must prepare a graduate who is competent in providing both primary and comprehensive preventive and therapeutic oral health care for infants and children through adolescence, including individuals with special health care needs.” We would expect that pediatric dental postgraduate programs may have a more structured curriculum to prepare residents to serve children and adolescents with ASD and/or ADHD, which can be adapted to orthodontic postgraduate training programs. However, the facts are disappointing as children with autism continually report more difficulty finding a pediatric dentist willing to provide care [39, 40]. The urgent training needs of pediatric dentistry residents to provide oral health care to children with SHCN, especially those with ASD, led HRSA to award funding to Nova Southeastern University College of Dentistry in 2010 to expand pediatric dental residency training by providing a focus on SHCN with an emphasis on ASD. The curriculum design as well as the outcome of this program were recently reported in 2020 [41]. Since then, no report could be found regarding the education status of pediatric dentistry programs on the topic of care for children with ASD. It would be interesting to see if and how the curriculum set up by the Nova Southeastern University College of Dentistry has been successfully adapted by other pediatric dentistry programs.

In addition, while both pediatric dentistry and orthodontics provide dental care to children with ASD and/or ADHD, there are some fundamental differences in the dental care provided by these two specialties that prevent orthodontic educators from directly adapting the curriculum set up by pediatric dentistry educators on the topic of providing dental care to patients with ASD and/or ADHD. First of all, almost all pediatric dentistry programs are equipped with general anesthesia/sedation stations since general anesthesia/sedation is frequently used to manage children due to extensive treatment needs, acute situational anxiety, uncooperative age‐appropriate behavior, limited cognitive functioning, physical disability, or medical conditions to complete dental treatment in a safe and humane fashion [42]. Therefore, the use of general anesthesia/sedation as needed for the pediatric dental care is endorsed by the AAPD [43]. However, orthodontic care rarely requires the use of general anesthesia/sedation. If there is a need, the orthodontic procedure is typically combined with other procedures such as extraction, endodontic treatment, or surgical exposure of an impacted tooth [44]. Thus, orthodontic clinics are rarely equipped with general anesthesia/sedation, and orthodontic postgraduate trainees are less exposed to clinical settings with general anesthesia/sedation compared to pediatric dentistry postgraduate trainees. Second, the routine dental care provided by pediatric dentists is normally every six months, while the frequency for office visits for active orthodontic care is normally every month. Although increased frequency of dental visits allows for regular exposure to the dental environment, which could potentially help children with ASD become more comfortable and familiar with the setting, the discomfort caused by orthodontic appliance adjustments experienced consistently on a monthly basis may lead to increased fear and resistance. In addition, while sedation can be considered when performing the most difficult procedures, such as full‐mouth orthodontic appliance bonding, it should be undertaken on the condition that the patient has already proven to be able to undergo the monthly appliance adjustment appointments with behavior management techniques alone [45]. Thus, the high frequency of appointments makes orthodontic care hold a larger demand for the patients and the caregivers’ willingness to cooperate when compared to pediatric dental care. Lastly, for most pediatric dental care, no appliances will be left in patients’ mouths after the dental visit, except for restorations that can be polished to create a smooth surface that makes it easier for the patients and caregivers to perform daily home dental care. In contrast, comprehensive orthodontic care normally requires fixed appliances that are cemented on the tooth surface. The bulkiness and rough surface of the orthodontic appliances significantly increases the discomfort level not only during the clinical visit, but also throughout the orthodontic treatment period while the appliance remains in the patient's mouth. In addition, orthodontic appliance maintenance is crucial. This includes properly wearing removable appliances at home, as well as preventing patients from manipulating the fixed appliances and arch wires, which could cause broken brackets and/or arch wires due to repetitive behavior—a core symptom of ASD [46]. Thus, we cannot rely on directly implementing the curriculum and experiences of other specialties to set up a curriculum that is specific to orthodontic care for patients with ASD and/or ADHD.

Every individual has their own unique preferences and tolerance levels, and we are not advocating for one universal treatment strategy that may be used to treat all patients with ASD and/or ADHD. It is also worth noting that ASD and/or ADHD are broad‐spectrum neurodevelopmental disorders that require individualized management plans. A single protocol does not apply to all patients due to the diversity of symptom presentations, the potential for co‐occurring conditions, and the individual's specific needs and strengths [5, 47]. Instead, we hope to bring attention to the current status, absence, and discrepancies of postgraduate orthodontic programs education on this topic and suggest the benefit of increased curriculum content on treating patients with ASD and/or ADHD. Since there is not enough clinical evidence suggesting the superiority of one method over another, it may be challenging to create standardized orthodontic guidelines on this topic. However, teaching common dental and behavioral characteristics associated with ASD and/or ADHD and techniques for effective treatment management can aid orthodontic residents in better accommodating this patient population. A close collaboration between orthodontists, pediatricians, psychologists, psychiatrists, and neurologists is needed to set up guidelines for the care of this patient population, as well as to set up the curriculum standards for both didactic and clinical trainings for orthodontic postgraduate programs.

Some limitations of this study include a small sample size of 29 respondents. In addition, since we only included programs in the United States and Canada, these results can only be generalized to programs within North America. It is worth noting that the respondents were not evenly distributed among regional constituent groups. For instance, more responses were received from the Southern Association of Orthodontists, Middle Atlantic Society of Orthodontists, and Northeastern Society of Orthodontists groups compared to the Great Lakes Associations of Orthodontists and Rocky Mountain Society of Orthodontists groups. This regionally uneven distribution may introduce certain location‐based bias into the current results, and may also represent the variation in interest levels of the program directors/department chairs on this ASD and/or ADHD related topic. In the future, a broader‐reaching study can be done to investigate the education status of other postgraduate programs in different countries, since the increase in the prevalence of ASD and ADHD is observed globally [2, 3]. It would also be beneficial to gain a more detailed understanding of curriculum content and compare which learning strategies residents feel are most practical to inform the topics and style of training that could be included in guidelines for orthodontic training on treating patients with ASD and/or ADHD. Expanding the survey to include the patients and caregivers in the clinic may also help understand the patients’ perspective on clinical care. Nevertheless, while didactic curriculum content is relatively easy to standardize by referring to textbooks and published guidelines, clinical implementation could be limited by facility resources, availability of qualified faculty members, and accessibility of the patient population. The current study did not collect information on whether the respondent programs were hospital‐based program or university‐affiliated program. Thus, we could not perform comparison analyses to see if one type of program has more clinical exposure and/or more well‐structured teaching resources compared to the other. Identifying and eliminating the obstacles of the clinical implementation for didactic curriculum content are critical steps for future studies to implement proper clinical training for the postgraduate orthodontic residents.

## 5 Conclusion

As diagnoses of ASD and/or ADHD are becoming more prevalent, so are the number of patients with these conditions who are entering the orthodontic clinic. At this time, there does not seem to be recent literature informing the status of orthodontic postgraduate education on treating patients with ASD and/or ADHD. The findings of this study indicate that for both clinical experience and didactic coursework, there is limited consistency and standardization in the current education provided by postgraduate orthodontic programs in North America on the topic of treating patients with ASD and/or ADHD. A systematic curriculum on treating these patients can help residents gain experience and be better prepared to treat this growing patient population.

## Author Contributions

**Nikki Wei**: methodology, software, formal analysis, investigation, data curation, writing – original draft. **Chun‐Hsi Chung**: validation, writing – review and editing. **Ilana Zinn**: validation, writing – review and editing. **Mark S. Wolff**: conceptualization, writing – review and editing. **Chenshuang Li**: conceptualization, methodology, writing – review and editing, funding acquisition, supervision, project administration.

## Conflicts of Interest

The authors declare no conflicts of interest.
